# The impact of soil erosion on internal migration in China

**DOI:** 10.1371/journal.pone.0215124

**Published:** 2019-04-16

**Authors:** Hua Zhang, Li Zhuang

**Affiliations:** 1 Beijing Key Laboratory of Environmental Remote Sensing and Digital Cities, Faculty of Geographical Science, Beijing Normal University, Beijing, China; 2 Institute for Urban and Environmental Studies, Chinese Academy of Social Sciences, Beijing, China; Institute for Advanced Sustainability Studies, GERMANY

## Abstract

The impact of environmental change on internal migration has received wide attention in recent years. Mass internal migration has been a significant economic and social phenomenon in China, and soil erosion is a major environmental problem that impacts sustainable socioeconomic development. This study aims to identify the impact of soil erosion on internal migration in China at the county level based on gravity model by analyzing related data, such as the sixth national population census data and the soil and water conservation survey data. The results of spatial overlay could not identify an obvious relationship between soil erosion and net outmigration in China. The traditional gravity model of migration is modified to analyze the impact of soil erosion on net outmigration while other variables are controlled. The results indicate that only serious soil erosion increases the possibility of outmigration and that the impact is considerably higher in agricultural counties than in non-agricultural counties. In general, the impact of soil erosion on internal migration is far less than the impact of socioeconomic factors.

## Introduction

As global environmental change has accelerated, its impact on internal migration has attracted great attention from researchers and policy makers [1]. Because it is an interdisciplinary research topic involving both natural and social sciences, great progress has been made from the viewpoints of demography, sociology, anthropology, geography, etc. However, a common understanding of many issues in this field has rarely been reached, and some opinions are even contradictory. For instance, the definitions of terms regarding the migration caused by environmental change have not been settled. Terms such as “environmental refugees,” “environmental migration,” and “eco-migration” frequently have appeared in the literature and sparked controversies [2–7]. Two distinct viewpoints exist with regard to the estimation of the impact of environmental change on internal migration: the maximalists and the minimalists [8]. The maximalists believe that environmental degradation causes mass internal migration, whereas the minimalists hold that the opinions of the maximalists are based on conjecture and lack sufficient empirical evidence. The minimalists view environmental change as a background variable, but it is difficult to reach a clear conclusion owing to the difficulty in analysis and the lack of evidence. There is still no final conclusion on how environmental change impacts internal migration. Taking drought as an example, many cases show that internal migration is a highly possible response to drought, whereas other studies reach the opposite conclusion that drought has scarcely any impact on internal migration [9–12]. In addition, there are disputes over which groups of humans are more vulnerable to environmental change. Chan showed that the rich may migrate, but the poor have lower migration capability owing to their inability to afford the migration costs [13]. However, Morrow-Jones showed that the lower socioeconomic class will most likely migrate after natural disasters, whereas the rich have access to more countermeasures to avoid the impacts of disasters, suffer less from disasters and more easily rebuild their homes [14]. The reason that it is difficult to reach common agreement on this issue is probably due to the complexity of migration decision making. On the one hand, there is a lack of appropriate data and methods to measure environmental change and internal migration; on the other hand, it is very difficult to separate environmental factors from socioeconomic factors that may also impact migration [12].

Compared to the research on internal migration, attention has just begun to be paid to the impact of environmental change on internal migration. Previous studies of this topic focused mainly on Africa and Southeast Asia, and very few focused on China. In China, soil erosion has become the top environmental problem that impacts sustainable socioeconomic development [12]. Soil erosion not only reduces the quality of cultivated land but also degrades the environmental conditions; intensifies the magnitude of flooding and drought; and has become the critical issue affecting the population, resources, and environment of China.

Rapidly expanding industrialization and urbanization also contribute to mass migration in China. Previous studies of internal migration in China were concerned mainly with inter-province migration and the patterns, features, and influencing factors of such large-scale migrations [15–19]. The impact of environmental change on internal migration in China is less discussed by previous studies than economic, social, traffic, and institutional factors. In short, it is still unclear whether and to what extent environmental factors, particularly soil erosion, drive migration in China.

This paper aims to identify the impact of soil erosion on internal migration in China at the county level. Therefore, the spatial distribution of soil erosion severity and net outmigration were investigated with the use of data from the national survey of soil and water conservation and the national population census. Then, the correlation between soil erosion severity and net migration was analyzed through spatial overlay. Finally, the modified gravity model of migration was used to separate soil erosion from other factors influencing internal migration so that the impact of soil erosion on internal migration in China could be scientifically analyzed.

The remainder of this paper is organized as follows. The next section describes the methods and data. The third section shows the spatial distribution of net outmigration and soil erosion. We analyze the impact of soil erosion on outmigration based on the modified gravity model in the fourth section, and conclude the paper by summarizing the conclusions finally.

## Materials and methods

### Spatial scale and data sources

One of the challenges in the study of the relationship between environmental change and internal migration is to obtain appropriate environmental and demographical data. Owing to the fact that huge differences in the natural environment exist within a given province, the use of the province as a basic unit is insufficient to reflect the impact of environmental change on internal migration. Clearly, such a spatial scale is too large for the current study. Considering the availability of data, the county is taken as the basic unit of this study to enable an accurate evaluation of the impact of soil erosion on internal migration in China.

To unify the spatial scales of environmental and socioeconomic data, this study takes the county-level administrative division in 2010 (excluding Hong Kong, Macau, and Taiwan) as the foundation and further processes the data based on the availability of data and the purpose of the study. Specifically, the municipal districts under a municipality directly governed by the central government and those under a prefecture-level city are integrated into one district. For instance, 14 municipal districts in Beijing are integrated into the Beijing urban district as a basic unit. Finally, 2282 county-level divisions are taken as basic units of the study. We then adjust the county boundaries of the map with a scale of 1:4000000 provided by the National Geomatics Center of China and obtain the vector data of the research unit boundaries.

In this study, the demographic data are from The 2010 Population Census of the People’s Republic of China for County, and other socioeconomic statistical data are from China Statistical Yearbook for Regional Economy 2011 and China County Statistical Yearbook 2011. Those statistical data are integrated and organized at the county level. The data on soil erosion are from the raster data of the soil and water conservation survey in the first national census for water in China, which was conducted in 2011; the spatial resolution of the data is 250 m.

### The measurement of internal migration

The China census data at the county level include population sizes only of in-migrants and do not include migration flows. Based on the definition of lifetime migration, this study tries to obtain migration data according to the difference between hukou location (place of registration) and place of residence. The hukou (household registration) system has a significant impact on internal migration in China. In addition to the difference between agricultural and non-agricultural accounts, the difference in hukou location also represents a difference in social welfare. Hukou location can be perceived as the place to which a person belongs [20]. If hukou location is taken as the origin of migration and place of residence is taken as the destination of migration, the difference between the two represents the occurrence of migration. Therefore, the difference in the size of the registered population and the size of the permanent population (DRP) can be used to study the features and the scale of net outmigration. The ratio of DRP to the size of the permanent population (DRP is negative) or the size of the registered population (DRP is positive) can be used to measure the intensity of net outmigration. If the DRP or net outmigration intensity of a county-level division is positive, the division is the area of net outmigration; if the converse is true, then the division is the area of net inmigration. It is important to note that according to the statistical methods of the census, the permanent population refers mainly to those who have lived in a local area for more than half a year; DRP is used to measure the size of inter-county migration and does not cover intra-county migration.

### Evaluation of soil erosion severity

In the survey of soil and water conservation in China, soil erosion is classified into three categories: water erosion, wind erosion, and freeze-thaw erosion. Soil erosion severity is classified into six degrees: very slight, slight, moderate, moderately severe, severe, and very severe. The severity of soil erosion at the county level is calculated with the soil erosion severity index (SESI) [21]. The equation is shown as follows:

$$SESI=\frac{\sum_{i=1}^{6}\left(A_{i}\times M_{i}\right)}{\sum_{i=1}^{6}A_{i}}$$ (1)

where i represents soil erosion severity degree and the value is from 1 to 6, ranging from very slight to very severe erosion; Ai represents the area with soil erosion at degree i; and Mi represents the weight of erosion at degree i. The value of Mi is evaluated in accordance with the soil erosion modulus of different erosion severity referenced in The Standards for Classification and Gradation of Soil Erosion (SL190-2007). The generated Mi is approximately 1/1000 of the average erosion modulus of the corresponding erosion severity. Specifically, the values of M1–M6 are 0, 1.5, 3, 6, 12, and 24, respectively. The resulting SESI score multiplied by 1000 is approximately the average of the soil erosion modulus of the research unit. Obviously, a higher SESI score represents higher soil erosion severity, and the index ranges from 0 to 24. When the soil erosion severity of a research unit is very low, the SESI score is 0, indicating that soil erosion is not serious. Conversely, when the soil erosion severity of a research unit is very severe, the SESI score is 24, indicating that soil erosion is the most serious.

### The modification of the gravity model

As mentioned previously, there are still many disputes over the impact of environmental change on internal migration, in part because of the difficulty in divorcing environmental factors from other factors. The research on internal migration can be traced back to Laws of Migration, issued by Ravenstein in 1885 [22]. In the paper, Ravenstein summarized seven laws of migration and discussed the motivations for and factors of migration. Herberle attributed the rural-urban migration of the labor force in Germany to the “push” of rural areas and the “pull” of urban areas [23]. Everett S. Lee expanded the push-pull factors into positive and negative factors at the origin and destination, intervening obstacles, and personal factors [24]. D. J. Bogue comprehensively and clearly formulated 12 push factors and 6 pull factors [25]. The push-pull theory, which states that migration is the result of many factors, including economic factors, can relevantly explain the causes of migration. However, the theory still has defects: push-pull factors are vague concepts, and it is difficult to determine the strength of pushing and pulling. Therefore, the theory can be used only for qualitative comparison and phenomena explanation. In the quantitative studies of internal migration, the gravity model plays the most significant role. Zipf replaced the masses of two objects with the populations of two areas and first introduced the gravity model into the research on migration [26]:

$$I_{ij}=K\frac{P_{i}P_{j}}{{D_{ij}}^{b}}$$ (2)

where ***I***_***ij***_ is the number of migrants between place i and place j; ***P***_***i***_ and ***P***_***j***_ are the population sizes of two areas, respectively; ***D***_***ij***_ is the distance between place i and place j; and K and b are constants.

The model shows that the number of migrants between two places is in direct proportion to the product of the population sizes of the two places and is in inverse proportion to the distance between the two places. The model is simple and straightforward; thus, it is widely used. Although the gravity model can describe migration well, it is criticized because it cannot explain the causes and motivations of migration [27]. To compensate for the defect, researchers introduced into their studies socioeconomic variables that may impact migration, such as income, employment, education, and migration stock of the places of origin and destination [28–31]. Considering different push and pull factors, the gravity model of internal migration can be summarized as follows:

$$I_{ij}=KR_{i}A_{j}f(D_{ij})$$ (3)

where ***R***_***i***_ and ***A***_***j***_ are the repulsion (push) of the place of origin i and the attraction (pull) of the place of destination j, respectively, inclusive of a series of socioeconomic variables that may impact migration, and ***f***(***D***_***ij***_) is the distance attenuation function.

Because it is difficult to obtain complete data on migration flows between the research units at the county level, and the objective of this study is to reveal whether soil erosion pushes the outmigration from the place of origin, this study focuses only on outmigration rather than the origin-destination matrix used in conventional studies. By summing j concurrently at both sides of Eq (3), the gravity model of outmigrants can be obtained as follows:

$$I_{i}=\sum_{j}I_{ij}=KR_{i}\sum_{j}A_{j}f(D_{ij})$$ (4)

where ***I***_***i***_ is the volume of outmigration from origin i. A greater pull force that attracts migrants to a destination may lead to more migrants. Therefore, the actual number of inmigrants of destination j can represent the strength of the pull with which place j attracts migrants from other places. In other words, the number of inmigrants of destination j can replace ***A***_***j***_. Thus, the gravity model of outmigrants is revised as follows:

$$I_{i}=KR_{i}\sum_{j}{IM}_{j}f(D_{ij})$$ (5)

where ***IM***_***j***_ is the total number of inmigrants of destination j and represents the pull with which place j attracts migrants from other places and ∑_***j***_***IM***_***j***_***f***(***D***_***ij***_) is the strength of the pull with which other places attract the migrants of origin i and can be viewed as the outmigration potential of origin i, which is similar to the notion of population potential [32]. Suppose that ***OP***_***i***_ = ∑_***j***_***IM***_***j***_***f***(***D***_***ij***_); if S factors pull internal migration, then

$$I_{i}=K∙\prod_{n=1}^{S}{R_{i,n}}^{\alpha_{1,n}}∙{{OP}_{i}}^{\alpha_{2}}$$ (6)

where ***R***_***i*,*n***_ is the nth variable that pushes outmigration from origin i, and ***α***_**1,*n***_ and α_**2**_ are to-be-estimated parameters. Taking the logarithm of both sides of the equation, the gravity model of outmigrants can be linearized as follows:

$$\ln\;I_{i}=\alpha_{0}+\sum_{n=1}^{S}\alpha_{1,n}\;\ln\;R_{i,n}+\alpha_{2}\;\ln\;{OP}_{i}$$ (7)

where ***α***_**0**_ = ln ***K***. The volume of outmigrants from the place of origin is determined by the push of the place of origin and the outmigration potential (the strength of pull exerted by other places on the place of origin). By examining the values of ***α***_**1**_ and ***α***_**2**_ and performing a significance test, whether and how the corresponding factors impact migration can be revealed after the effects of other variables are controlled.

## The spatial overlay analysis of outmigration and soil erosion at the county level

### The spatial distribution of net migration

With the rapid development of industrialization and urbanization, mass migration has become a common and significant social phenomenon in China. According to the sixth national census, conducted in 2010, the number of inter-county migrants calculated by the difference between registered population and permanent population exceeded 170 million, accounting for 12.8% of the total population of China. The number of intra-province migrants reached 84,689,000, which is 49.65% of the total number of inter-county migrants and is close to the number of inter-province migrants. Regarding the type of migration, 617 counties have net inmigration, accounting for 27.04% of the total number of county-level divisions, whereas 1,665 counties have net outmigration, accounting for 72.96% of the total number of county-level divisions. The number of places with net outmigration is 2.7 times the number of places with net inmigration.

**Fig 1:**
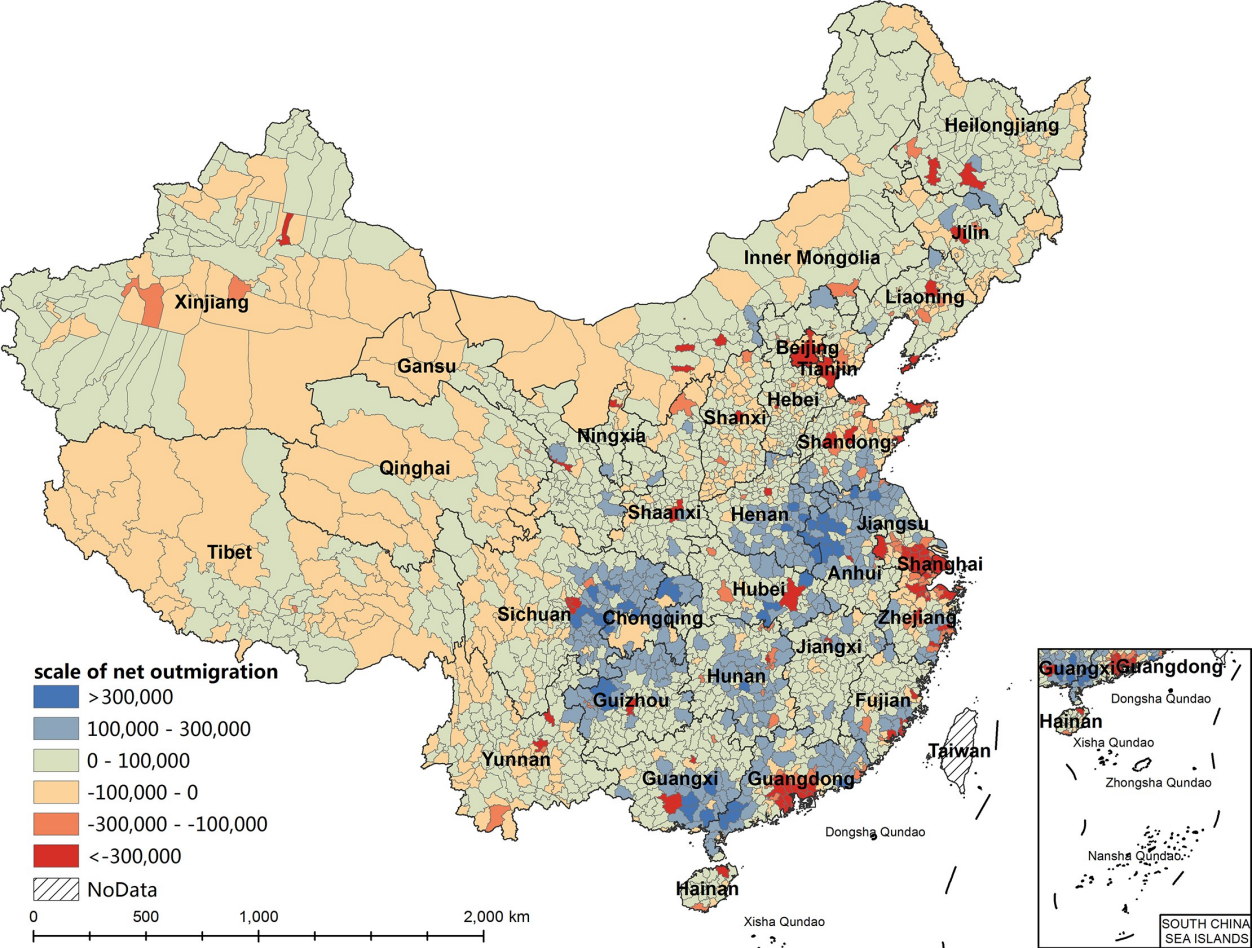
The spatial distribution of the scale of net outmigration at the county level in China, 2010.

**Fig 2:**
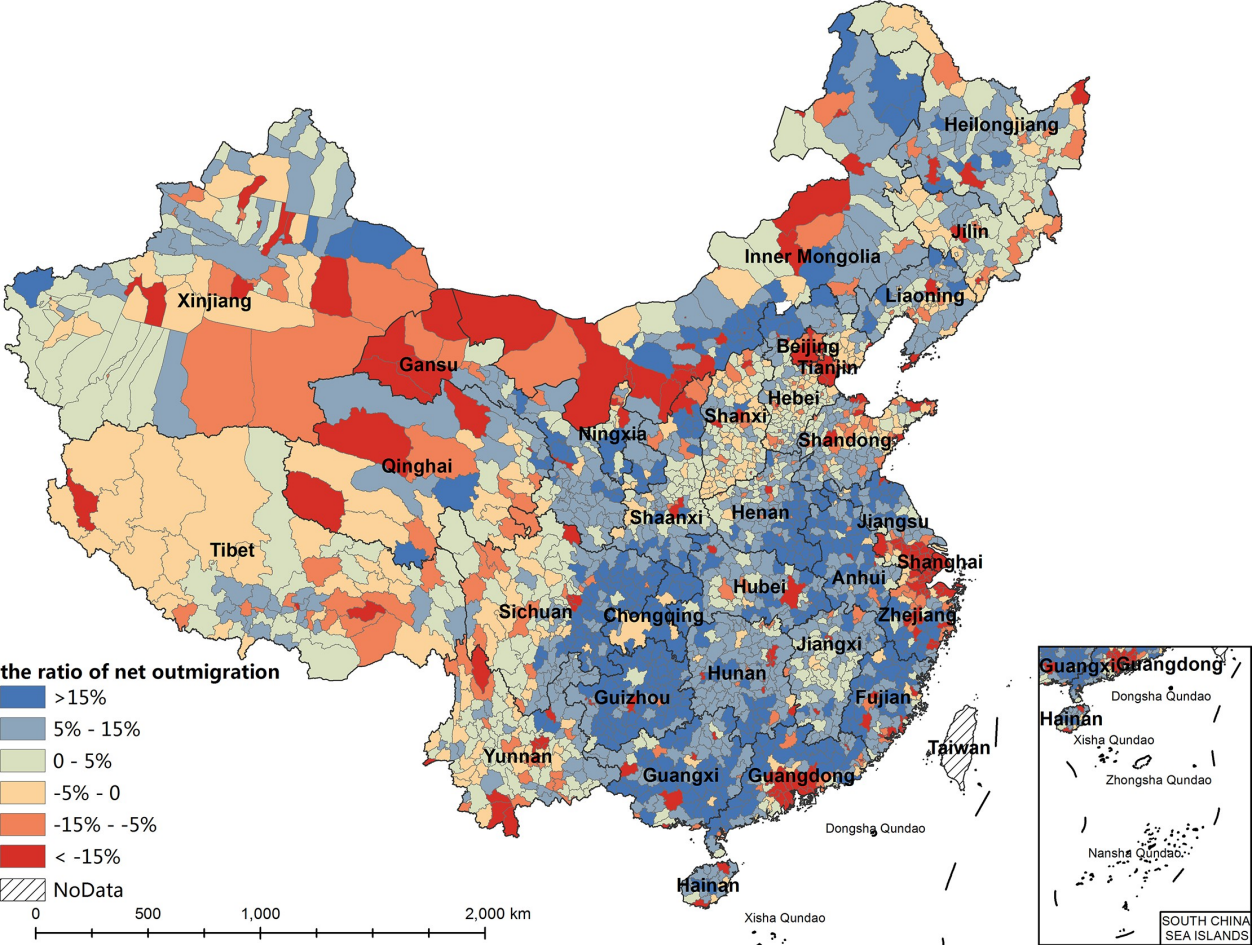
The spatial distribution of ratio of net outmigration based on population at the county level in China, 2010.

Regarding the spatial distribution of migration (Figs 1 and 2), the net inmigration counties are distributed mainly in coastal areas of eastern China; border regions in western China, such as Xinjiang and Inner Mongolia; and municipal districts in the capitals of other provinces. The counties with more than 300,000 net inmigrants are concentrated in the urban agglomerations of the Pearl River Delta, the Yangtze River Delta, and the Beijing-Tianjin-Hebei region. The net inmigration area in western China also has a high net inmigration ratio, however, this ratio is due mainly to the small population size of the area. The number of inmigrants of most net inmigration counties in western China is within 100,000, lower than the number in eastern China. The counties with net outmigration are distributed mainly in the densely populated areas in central and western China and rural areas of eastern China. The counties with more than 300,000 net outmigrants are concentrated mainly in three areas: from eastern Sichuan to northwestern Guizhou and eastern Chongqing, from southeastern Henan to northwestern Anhui, and from southeastern Guangxi to southwestern Guangdong.

### The spatial distribution of soil erosion

Excessive soil erosion is a worldwide social and economic problem and is one of the environmental problems that require attention in China [33, 34]. The soil and water loss problem in China is one of the most serious in the world because of the great variety of topography, changeable and diversified climate, and extremely high level of human activity. According to the report of the soil and water conservation survey in the first national census for water in China, the total area of soil erosion in China is 2,949,100 km^2^, accounting for 31.12% of the total surveyed area. The area of water erosion is 1,293,200 km^2^, accounting for 13.65% of the total surveyed area, and the area of wind erosion is 1,655,900 km^2^, accounting for 17.47% of the total surveyed area. In addition, the area of freeze-thaw erosion is 661,000 km^2^.

**Fig 3:**
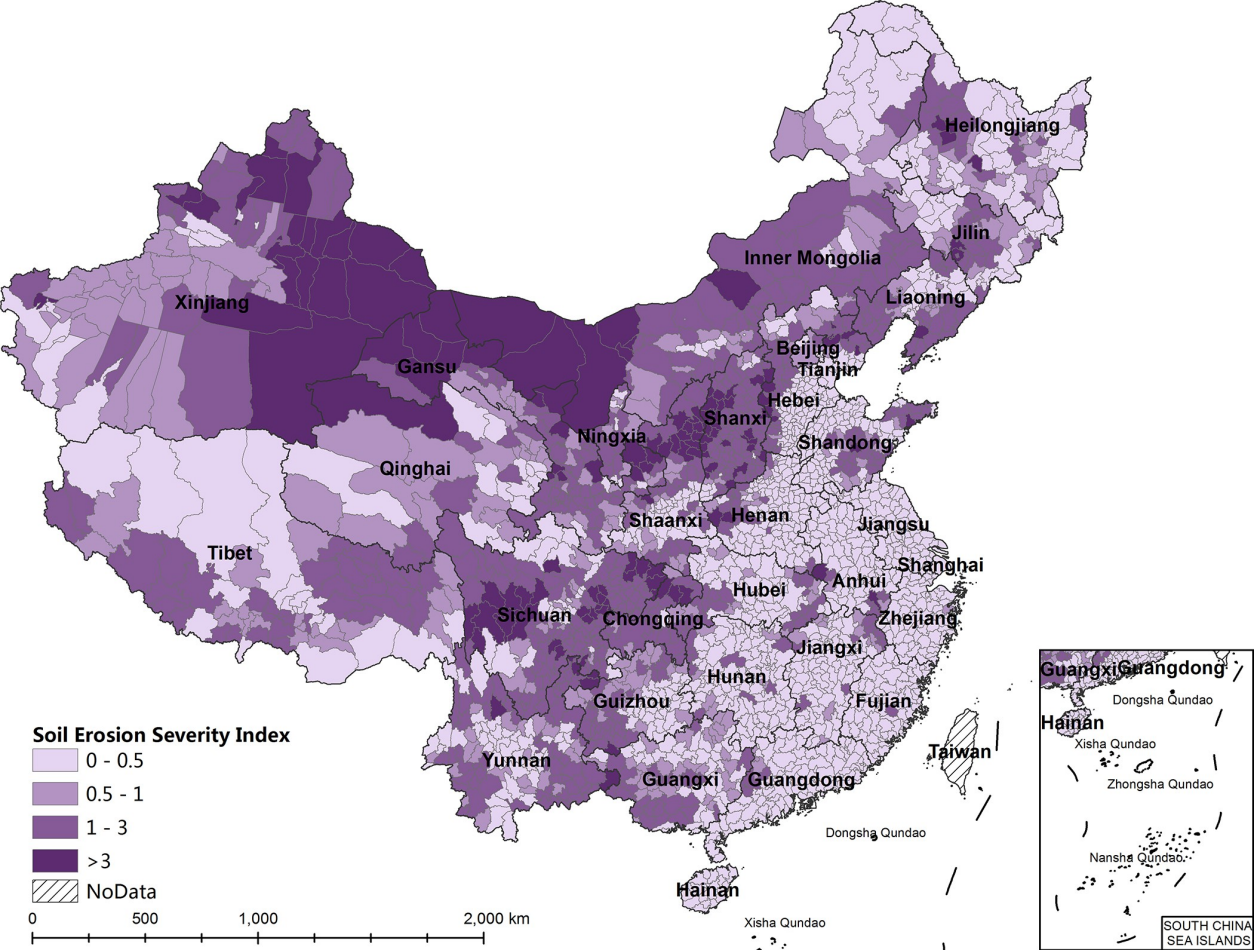
The spatial distribution of soil erosion severity at the county level in China, 2010.

As mentioned above, the SESI is approximately 1/1000 of the average of the soil erosion modulus of the research unit. Therefore, from the standpoint of soil erosion severity, a SESI score lower than 0.5 may indicate that no serious soil erosion exists; a SESI score between 0.5 and 1 indicates that the soil erosion severity is slight and that there is less serious soil erosion in the corresponding areas; a SESI score between 1 and 3 indicates serious soil erosion; and a SESI score higher than 3 indicates very serious soil erosion [21]. Therefore, the research units are divided into four classes: not serious, less serious, serious, and very serious. Among them, 642 counties have serious soil erosion, accounting for 28.13% of all units, and 144 counties have very serious soil erosion, accounting for 6.31% of all units. Regarding the spatial distribution (Fig 3), western China suffers the most serious soil erosion because 61.53% of the counties with serious soil erosion and 67.36% of those with very serious soil erosion are distributed there. Soil erosion in central China and northeastern China is less serious than in western China, and soil erosion is lightest in eastern China. Wind erosion is distributed mainly in Xinjiang, Inner Mongolia, Gansu, and Qinghai; water erosion is distributed mainly in Sichuan, Chongqing, Yunnan, Guizhou, Shanxi, Shaanxi, Gansu, Ningxia, and Liaoning; and freeze-thaw erosion is distributed mainly in Tibet.

### The spatial matching relationship between net outmigration and soil erosion

Soil erosion destroys soil structure, reduces agricultural productivity, causes deposition of rivers and lakes, intensifies drought/flood, and degrades the environmental conditions, thus limiting the sustainable development of the national economy and the improvement of people’s livelihoods. Serious erosion not only causes the infertility of cultivated land and the poverty of local people but also causes desertification and destroys the basic conditions of human existence [35]. Although the methods and standards used to measure soil erosion are not exactly the same, all previous studies have indicated an obvious relationship between soil erosion and poverty in China [36, 37]. Poverty caused by environmental factors may motivate people to migrate to developed areas to make a living, but the poor may also be trapped in the harsh environment because of their inability to afford the necessary cost of migration [38]. Environmental change does not necessarily lead to internal migration, and the relationship between environmental change and migration is still an important research direction. Soil erosion is one of the most important environmental problems in China, and its impact on internal migration is worth further exploration.

**Fig 4:**
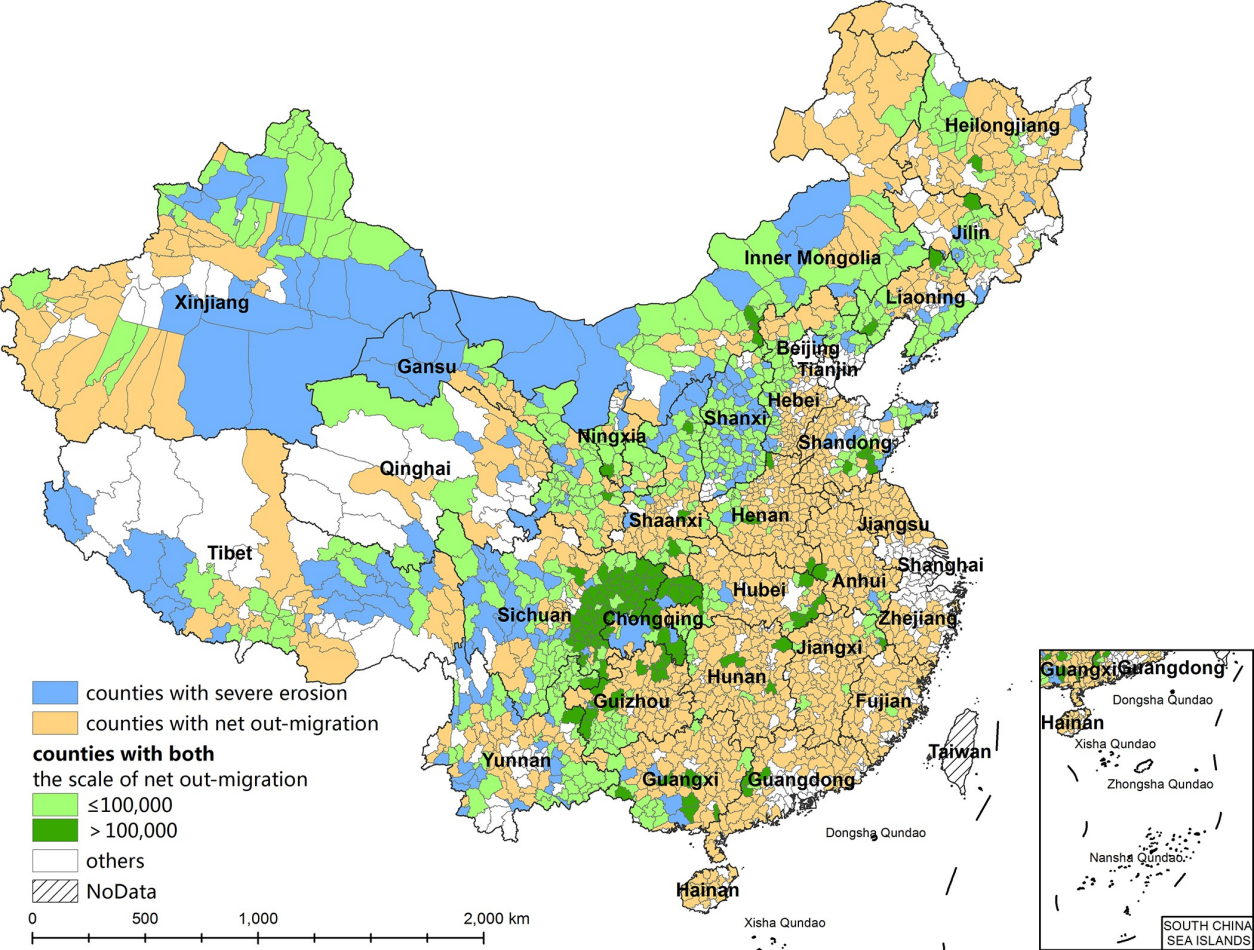
The spatial matching relationship between counties with severe erosion and counties with net outmigration in China.

**Fig 5:**
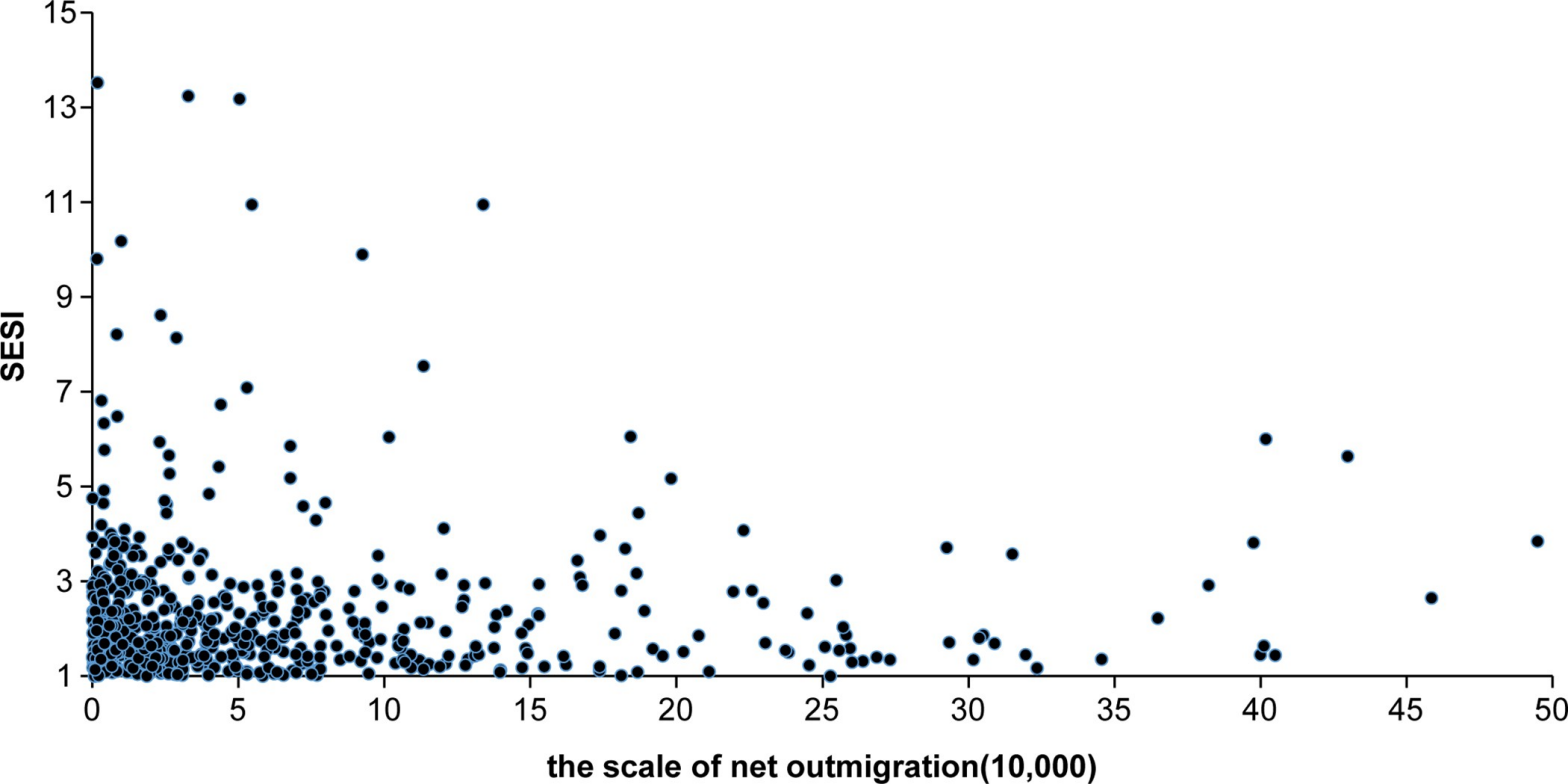
Scatterplot of the scale of net outmigration versus the soil erosion severity index (SESI) of counties with both.

**Fig 6:**
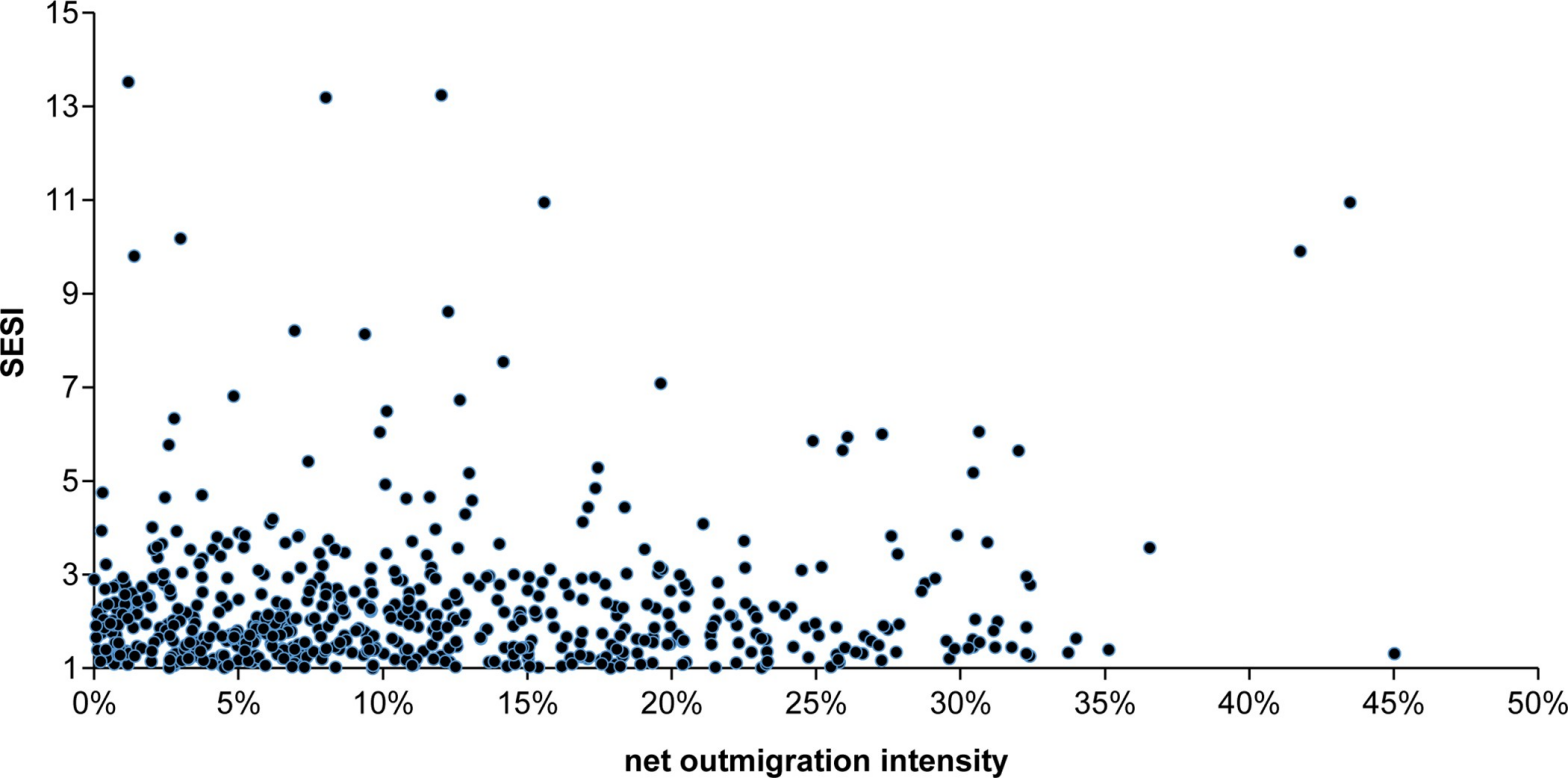
Scatterplot of the net outmigration intensity versus the soil erosion severity index (SESI) of counties with both.

Spatial overlap between the counties suffering serious soil erosion (including those with very serious soil erosion) and those with net outmigration (Fig 4) shows that 570 county-level divisions have both serious/very serious soil erosion and net outmigration, accounting for 34.23% of the counties with net outmigration (1,665 counties) and 72.52% of the counties with serious/very serious soil erosion (786 counties). In other words, approximately 1/3 of counties with net outmigration also suffer serious/very serious soil erosion (Table 1). In addition, although three-quarters of the counties that suffer serious/very serious soil erosion also have net outmigration, the number of counties with more than 100,000 net outmigrants is only 115, less than 1/6 the number of counties that suffer serious/very serious soil erosion. Meanwhile, there is no linear relationship between net outmigration and soil erosion severity in terms of both migration scale and intensity (Figs 5 and 6). Higher soil erosion severity does not indicate a larger scale of net outmigration. This result indicates that soil erosion is just one of the factors influencing internal migration.

**Table 1:** Results of the spatial matching.

	The number of counties with net inmigration	The number of counties with net outmigration	Total
The number of counties suffering slight soil erosion	401	1095	1496
The number of counties suffering serious soil erosion	216	570	786
Total	617	1665	2282

Thus, from the perspective of spatial matching, the relationship between soil erosion and net outmigration is not obvious. On the one hand, net outmigration at the county level does not include intra-county migration and cannot fully reflect the impact of soil erosion on internal migration. On the other hand, the factors influencing migration decisions are highly complex. Whether migration will occur depends on the interactions of all the factors. Moreover, environmental change is usually related to socioeconomic factors and is not the only force driving internal migration. Therefore, the role of socioeconomic variables should be controlled by modeling to clarify whether soil erosion pushes outmigration.

## Quantitative analysis based on the modified gravity model

This analysis takes Eq (7) as a basic model and DRP as a dependent variable. It also separates the factors influencing outmigration into the push of origins and the pull of destinations. Considering the data availability, the explanatory variables are as follows.

1. The population size of the place of origin (P_***i***_). Population size is a typical variable in the migration gravity model. It is generally believed that with other conditions unchanged, the population sizes of both the place of origin and the place of destination are positively correlated with the scale of migration [39]. However, some researchers believe that population size reflects the roles of other determinants of migration, and it is very difficult to interpret the statistical associations between population size and gross migration rates [40]. Population size is related to economic opportunities caused by agglomeration economies; thus, people will move from less populated to more populated places. Meanwhile, the large size of the population at the place of origin might mean a rural population surplus, which will lead to outmigration [13]. However, in this study, the sample of the dependent variable is related to the places with net outmigration. It is obvious that the push of population size is higher than the pull, and the population size of the place of origin should thus be positively correlated with the scale of migration. In addition, the dependent variable is related to the scale of outmigration but not to the intensity. Therefore, the population size but not the population density was adopted as the independent variable. To avoid explaining past migrations with the current population size and to prevent the distortion of the sequential relation between migration and population size, the size of the registered population is used in this study instead of the size of the permanent population to reflect the original population size of the place of origin.
2. Per capita GDP of the place of origin (G_***i***_). The per capita GDP represents the level of economic development. A lower per capita GDP indicates less economic opportunity and more inclination to outmigration. Conversely, with the other conditions unchanged, a higher per capita GDP usually corresponds to a smaller volume of outmigrants. Because per capita GDP is used as a kind of proxy variable for the local economic development level, it is calculated using GDP divided by the size of the permanent population but not the size of the registered population.
3. SESI_***i***_. This method of measurement was mentioned above, and its natural exponential form is used in the model. A higher SESI score indicates more serious soil erosion in the research unit. If the regression coefficient is positive and significant, this finding indicates that soil erosion can stimulate outmigration from the county-level divisions in China.
4. Outmigration potential (OP_***i***_). Outmigration potential is the total of the ratio of the inmigration scale of other places and the distance attenuation function between other places and the place of origin; it represents the pull exerted by other places on the place of origin. With other conditions unchanged, higher outmigration potential indicates a larger number of outmigrants. The inmigration scale in this study is the number of inmigrants from other counties within the province added to the number from other provinces. The distance attenuation function is defined as the reciprocal of the distance cost in accordance with the classic population potential model. Distance cost is defined as the Euclidean distance between the geometric centers of the research units.

Thus, the gravity model of outmigrants concerned with soil erosion severity is obtained:

$$\ln\;I_{i}=\alpha_{0}+\alpha_{1}\;\ln\;P_{i}+\alpha_{2}\;\ln\;G_{i}+\alpha_{3}{SESI}_{i}+\alpha_{4}\;\ln\;{OP}_{i}$$ (8)

The model is used to test the impact of soil erosion on outmigration. As a result, the sample of the dependent variable is related only to the county-level divisions where the DRP is positive. The number of such divisions is 1,665. It is worth noting that all county-level divisions should be considered when calculating outmigration potential, and the formula is ${OP}_{i}=\sum_{j=1}^{2282}\frac{{IM}_{j}}{D_{ij}}$. According to the analysis made previously, ***α***_**1**_, ***α***_**3**_ and ***α***_**4**_ should be positive, and ***α***_**2**_ should be negative.

A regression analysis is conducted upon the modified gravity model M1 (Eq (8)), which considers soil erosion severity using a sample of 1,665 counties. The correlation coefficient between variables ranges from −0.397 to 0.085 in our sample, suggesting that multicollinearity is not a serious issue. The regression results are shown in Table 2. The fit of the regressions is generally good, with an adjusted R^2^ of 0.531. Meanwhile, all regression coefficients of the model have the expected signs. The standardized regression coefficient indicates that the population size of the place of origin is the most important variable that impacts outmigration, a finding that is consistent with the conclusions of other researchers regarding inter-province migration in China [16, 18]. The standardized regression coefficient of per capita GDP of the place of origin and that of outmigration potential are -0.092 and 0.089, respectively. There is only a small absolute difference between these two values. As such, a good economic development level in the place of origin will hinder outmigration, and the pull of other places will have a positive impact on outmigration. However, the impact of these two determinants is not as high as the push of the population size of the place of origin. The standardized regression coefficient of the SESI is the lowest and is not significant, indicating no obvious relationship between outmigration and soil erosion of the place of origin at the county level in China. Some international studies also show no obvious causal relationship between environmental factors and internal migration, and the impact of environmental factors on migration is weaker than the impact of socioeconomic factors [10, 41, 42].

**Table 2:** Regression results of the gravity model.

Variable^a^	M1		M2		M3		M4		M5	
	B^b^	Beta^c^	B	Beta	B	Beta	B	Beta	B	Beta
Constant	-8.181***		-8.185***		-8.203***		-8.801***		-8.581***	
lnP	1.312***	0.722	1.313***	0.722	1.313***	0.723	1.314***	0.723	1.341***	0.744
lnG	-0.247***	-0.092	-0.247***	-0.092	-0.246***	-0.092	-0.100**	-0.037	-0.119**	-0.042
lnOP	0.316***	0.089	0.317***	0.089	0.318***	0.089	0.296***	0.083	0.320***	0.090
SESI	0.019	0.018					0.037**	0.034	0.033*	0.030
D1			0.216**	0.034	0.221**	0.004				
D2					0.014	0.034				
DA							0.687***	0.135		
Number	1665		1665		1665		1665		1497	
Adjusted R^2^	0.531		0.532		0.532		0.546		0.558	

a: Dependent variable = ln(DRP).

b: Regression coefficient.

c: Standard regression coefficient.

*p < 0.1

**p < 0.05

***p < 0.01.

When considering the complexity of migration, this conclusion is acceptable. Currently, regional inequality and rural-urban economic differences in China are the major motives of migration. In contrast, even the SESI can reflect environmental conditions, which are not necessarily the determining factor of outmigration. In contrast to extreme natural disasters, gradual environmental change allows humans to determine how they will respond to it [43]. Migration is just one of the possibilities for humans to cope with and adapt to environmental changes. Different human groups have different responses when faced with the same environmental change, and some have higher tolerance, whereas others are more vulnerable. Likewise, different levels of environmental degradation may lead to different migration results. People might not make a migration decision unless a certain threshold, or “tipping point,” is reached. Therefore, we introduce the dummy variable D1i to denote the counties suffering very serious soil erosion, taking the value 1 when the SESI score is greater than or equal to 3 and the value 0 otherwise, and replace SESIi in model M1 with D1i. Finally, model M2 is obtained. Regression results (Table 2) show that the coefficient of D1i is significant at the 5% level. With other conditions unchanged, the scale of net outmigration from the counties suffering very serious soil erosion is 24.11% higher than that of other counties. Further, another dummy variable, D2i, is introduced into the model to denote the counties suffering serious soil erosion, taking the value 1 when the SESI score is less than 3 and greater than or equal to 1 and the value 0 otherwise. Therefore, the sample counties are separated into three types in accordance with soil erosion severity: counties suffering very serious soil erosion, counties suffering serious soil erosion, and counties suffering less serious soil erosion. The regression coefficient D2i is not significant in the regression result of the corresponding model (M3), indicating that there is no obvious causal relationship between serious soil erosion and outmigration. This fact further proves that outmigration may not be motivated unless soil erosion is serious to a certain degree. The majority of migrants interviewed in the EACH-FOR project indicated that if the environment had affected a decision to migrate, it was most often because environmental changes had made it difficult for the individual or family to earn a living [44]. That is to say, there is a threshold of severity above which soil erosion becomes a significant influence on outmigration, and this finding is consistent with McLeman, R [45] and Nawrotzki, R.J. [46].

Environmental changes have a direct impact on migration through changes to environmental drivers and have an indirect impact on migration through changes to other drivers. For instance, they may affect economic drivers of migration by impacting agricultural productivity and rural livelihood [47]. Compared to the livelihood of urban citizens who work in non-agricultural industries, rural livelihood directly relies on the natural environment (land, soil, forest, and water resources). Degradation of land resources will undermine the sustainability of rural production systems and livelihoods, which is likely to stimulate rural outmigration once a threshold, or tipping point, is reached [11]. Therefore, soil erosion most likely impacts areas with agriculture as the major industry. The damage of soil structure will cause the decline of agricultural productivity and induce outmigration. To prove the assumption, a dummy variable (DAi) for agricultural counties is introduced into model M1, and the new model M4 is obtained. In 2010, the added value of primary industry accounted for 10.1% of GDP in China, and DAi takes the value 1 when the percentage of the primary industry exceeds the average level (10.1%) and the value 0 otherwise. Regression results show that after the addition of DAi, the standardized regression coefficient of the SESI is significant at the 5% level, and its absolute value is near that of the per capita GDP of the place of origin. With other conditions unchanged, the scale of net outmigration from agricultural counties is 98.77% higher than that from non-agricultural counties. Thus, it is obvious that soil erosion will motivate outmigration in agriculture-dominated areas. The soil erosion severity is in direct proportion to the outmigration scale, and its impact is near the impact of the local economic development level. Taking 1,497 agricultural counties as samples and performing regression analysis on model M1 results in the same conclusion (M5). There is no clear difference between the standardized regression coefficient for the SESI and that for the per capita GDP of the place of origin, and both are significant at the 10% and 5% levels respectively.

In addition, in the models, the absolute values of the standardized regression coefficient of the population size of the place of origin all rank highest among the variables, higher than that of the per capita GDP and the outmigration potential of the place of origin, and the value of soil erosion severity ranks lowest. Hence, the population size of the place of origin strongly pushes county-level migration and has a higher impact on internal migration than the economic development level and the pull of the destination. This fact is consistent with the conclusions of previous studies on inter-province migration in China [16, 18, 48]. Soil erosion will not push outmigration unless it is serious to a certain extent or occurs in agricultural counties, and its impact on outmigration is lower than the impact of socioeconomic drivers.

## Conclusions

This paper discusses the impact of soil erosion on internal migration in China. In accordance with the data from the soil and water conservation survey in the first national census for water in China and the sixth China population census, DRP and the SESI are used to measure the net outmigration and soil erosion severity of the county-level divisions, respectively. On this basis, spatial overlap is used to analyze the spatial matching relationship between the counties that suffer serious soil erosion and those with net outmigration. The conventional gravity model of internal migration is modified to apply to the study of migration at the county level and is used to analyze the impact of soil erosion on outmigration while other variables are controlled.

In contrast to micro-studies on the motives and decision making for migration from the standpoint of individuals or households, studies at the county level are more at a macro level, focusing mainly on places of origin and destination. Although county-level studies are more suitable for analyzing the impact of environmental changes, the amount of socioeconomic data that impact migration available for county-level studies is less than that available for province-level studies. In addition to soil erosion, other environmental problems, such as land salinization, water shortage, and pollution, also impact internal migration. In addition to the SESI, only a few variables that impact migration are considered in the models. This restriction may be one reason that the explanatory power of the gravity model is not very strong. Furthermore, the Euclidean distance between the geometric centers possibly cannot reflect the real distance cost of inter-county migration, and the form of the distance attenuation function *f*(*D*_*ij*_) also requires further discussion. Despite the above-mentioned defects due to the complexity of migration decision making and the limitations of the data, the following meaningful conclusions are reached.

1. The counties with net outmigration are distributed both in densely populated areas in central and western China and in rural areas of eastern China. Soil erosion severity in western China is higher than that in central and eastern China. The spatial matching relationship between net outmigration and soil erosion shows that soil erosion in the counties with net outmigration is not necessarily serious, whereas the counties suffering serious soil erosion usually have net outmigration. However, regarding the scale of net outmigration, there is no obvious positive correlation between soil erosion severity and migration scale. It is difficult to ascertain the relationship between soil erosion and internal migration only by spatial overlay of the county-level divisions.
2. Regression results of the modified gravity model of migration show that soil erosion will not push outmigration unless it is serious to a certain extent with other conditions unchanged. For agricultural counties, the outmigration scale is in direct proportion to soil erosion severity, and the impact of soil erosion on outmigration from agricultural counties is higher than that from non-agricultural counties. However, in general, the impact of soil erosion on internal migration is far less than the impact of socioeconomic drivers such as population size and economic development level.
